# Biological properties of the BCL-2 family protein BCL-RAMBO, which regulates apoptosis, mitochondrial fragmentation, and mitophagy

**DOI:** 10.3389/fcell.2022.1065702

**Published:** 2022-12-16

**Authors:** Takao Kataoka

**Affiliations:** ^1^ Department of Applied Biology, Kyoto Institute of Technology, Kyoto, Japan; ^2^ Biomedical Research Center, Kyoto Institute of Technology, Kyoto, Japan

**Keywords:** BCL-RAMBO, BCL2L13, apoptosis, mitochondrial fragmentation, mitophagy, cell death, phosphorylation, miRNAs

## Abstract

Mitochondria play an essential role in the regulation of cellular stress responses, including cell death. Damaged mitochondria are removed by fission and fusion cycles and mitophagy, which counteract cell death. BCL-2 family proteins possess one to four BCL-2 homology domains and regulate apoptosis signaling at mitochondria. BCL-RAMBO, also known as BCL2-like 13 (BCL2L13), was initially identified as one of the BCL-2 family proteins inducing apoptosis. Mitophagy receptors recruit the ATG8 family proteins MAP1LC3/GABARAP *via* the MAP1LC3-interacting region (LIR) motif to initiate mitophagy. In addition to apoptosis, BCL-RAMBO has recently been identified as a mitophagy receptor that possesses the LIR motif and regulates mitochondrial fragmentation and mitophagy. In the 20 years since its discovery, many important findings on BCL-RAMBO have been increasingly reported. The biological properties of BCL-RAMBO are reviewed herein.

## Introduction

Mitochondria play an essential role in energy metabolism by producing ATP and many other metabolites, and also regulate cellular stress responses, such as cell death, autophagy, and inflammation (Green et al., 2011). Mitochondria harbor multiple components that transmit cell death signals and inflammatory signals (Gurung et al., 2015; Bock and Tait, 2020). Damaged mitochondria are disposed by a mitochondrial autophagy process (mitophagy), which counteracts cell death or inflammation (Green et al., 2011). Mitochondrial fission and fusion cycles play a critical role in quality control, which is linked to mitophagy (Youle and van der Bliek, 2012). Mitochondrial dysfunction causes many diseases, including neurodegenerative, cardiovascular, and metabolic disorders (Nunnari and Suomalainen, 2012). Mitochondria also regulate the initiation, growth, survival, and metastasis stages of tumorigenesis (Vyas et al., 2016). Therefore, a detailed understanding of the molecular mechanisms underlying mitophagy is important for therapeutic interventions. Mitophagy is initiated by two major pathways: the ubiquitin-dependent pathway and ubiquitin-independent pathway. PTEN-induced putative kinase 1 (PINK1) and the E3 ubiquitin ligase Parkin play a primary role in the regulation of ubiquitin-dependent mitophagy (Pickrell and Youle, 2015; McWilliams and Muqit, 2017). In contrast, ubiquitin-independent mitophagy is mediated by at least five mitophagy receptors anchored to mitochondrial outer membranes (MOM). In addition to apoptosis, the BCL-2 family protein BCL-RAMBO, also known as BCL2-like 13 (BCL2L13), has recently been identified as a mitophagy receptor that regulates mitochondrial fragmentation and mitophagy. The biological properties of BCL-RAMBO are reviewed herein.

### Regulation of cell death

**FIGURE 1:**
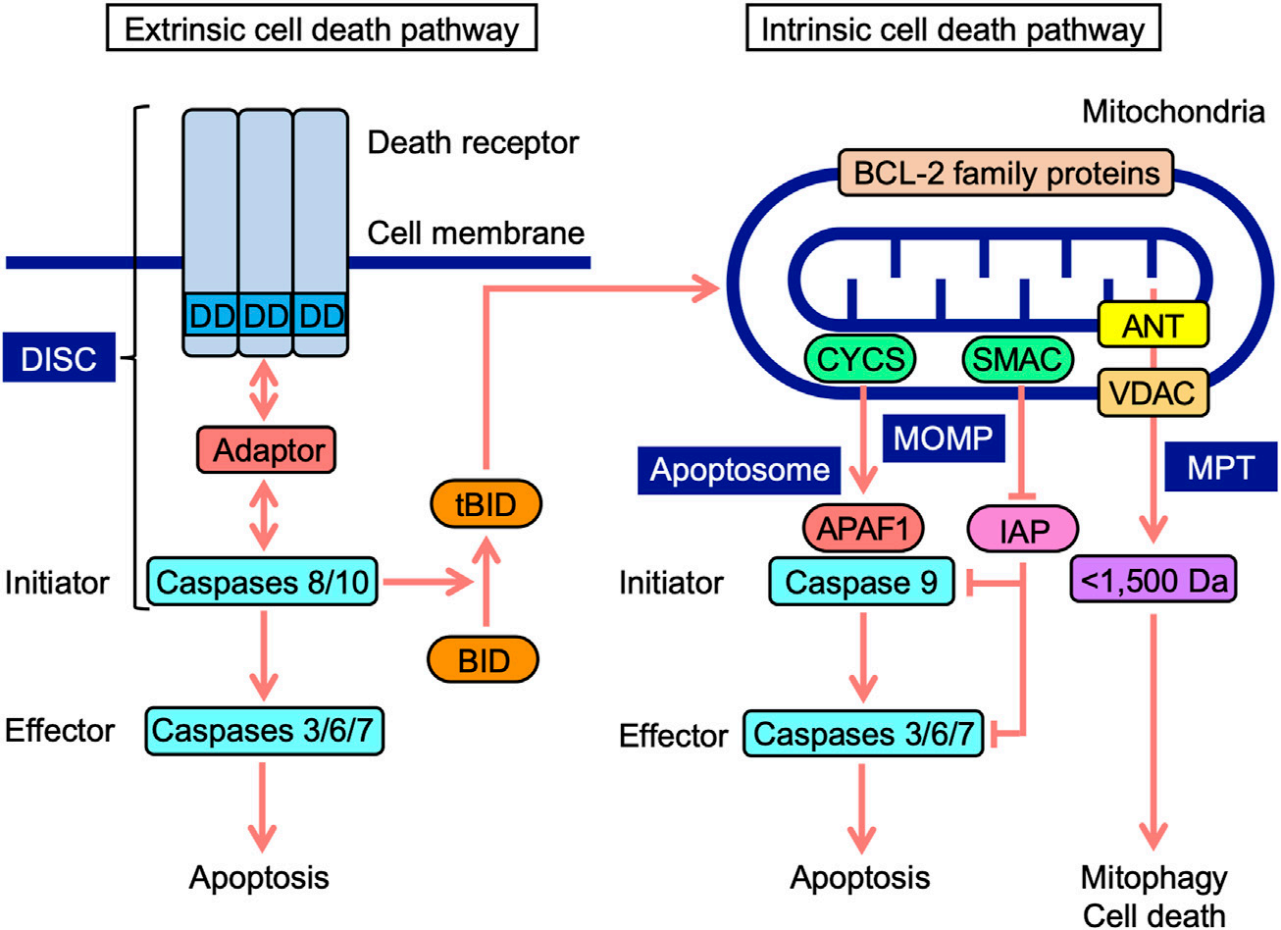
Extrinsic and intrinsic cell death signaling pathways. In the extrinsic pathway, death receptors, upon engagement with ligands, recruit adaptor proteins to their death domain (DD) and procaspases 8 and 10, leading to the formation of a death-inducing signaling complex (DISC). In the DISC, procaspases 8 and 10 are activated into initiator active caspases 8 and 10, which cleave procaspases 3, 6, and 7 to effector active caspases 3, 6, and 7, leading to apoptosis. Mitochondria harbor BCL-2 family proteins and VDAC on mitochondrial outer membranes, ANT on mitochondrial inner membranes, and CYCS and SMAC at the intermembrane space. In the intrinsic pathway, mitochondrial outer membrane permeability (MOMP) causes the release of CYCS and SMAC into the cytosol. CYCS induces complex formation consisting of APAF1 and procaspase 9 (apoptosome). In the apoptosome, procaspase 9 is activated to initiator active caspase 9, which cleaves procaspases 3, 6, and 7 to active caspases 3, 6, and 7, leading to apoptosis. SMAC antagonizes IAP proteins, which target caspases. Mitochondrial permeability transition (MPT) releases <1,500-Da solutes, which cause mitophagy and cell death. BH3-interacting domain death agonist (BID) is cleaved into truncated BID (tBID) by active caspases 8 and 10. tBID translocates to mitochondria and interacts with BCL-2 family proteins, triggering apoptosis signaling.

Cell death occurs in multiple forms, including apoptosis, autophagy-dependent cell death, and necroptosis (Tang et al., 2019). Apoptosis is mediated by caspases, which are divided into initiator, effector, and inflammatory caspases (Mcllwain et al., 2013; Man and Kanneganti, 2016). The cascade of caspase activation is triggered by the ligation of cell-surface death receptors and mitochondrial activation resulting in the release of their components (Green et al., 2014). Death receptors and mitochondria trigger signaling pathways that are mediated by different initiator caspases (Figure 1). In the extrinsic pathway, death receptors recruit multiple adaptor proteins and procaspases 8 and 10 in the death-inducing signaling complex, and this is followed by their activation (Lavrik and Krammer, 2012; Siegmund et al., 2017). Initiator active caspases 8 and 10 convert procaspases 3, 6, and 7 to effector active caspases by cleavage, leading to the limited proteolysis of many substrates and the execution of apoptosis (Julien and Wells, 2017). In addition to energy metabolism, mitochondria play an essential role in programmed cell death (Bock and Tait, 2020). In the intrinsic pathway upon apoptotic stimuli, mitochondrial outer membrane permeability (MOMP) causes the release of intermembrane space proteins, two of which are cytochrome *c* (CYCS) and second mitochondria-derived activator of caspase (SMAC) (Cosentino and García-Sáez, 2017; Kale et al., 2018; Kalkavan and Green, 2018). In mammals, CYCS induces the activation of procaspase 9 through the formation of apoptosomes in the cytosol (Bratton and Salvesen, 2010; Yuan and Akey, 2013). Inhibitor of apoptosis (IAP) proteins target caspases *via* multiple mechanisms, while SMAC antagonizes IAP proteins (Fulda and Vucic, 2012). In a similar manner to caspases 8 and 10, initiator active caspase 9 cleaves downstream procaspases 3, 6, and 7 into their active forms (Mcllwain et al., 2013). BCL-2 family proteins possess one to four BCL-2 homology (BH) domains (BH1, BH2, BH3, and BH4) and are divided into three groups: anti-apoptotic, pro-apoptotic multi-domain, and pro-apoptotic BH3-only proteins (Adams and Cory, 2018; Singh et al., 2019; Warren et al., 2019). BCL-2 family proteins regulate apoptosis signaling by interacting with pro- and anti-apoptotic proteins at mitochondria (Adams and Cory, 2018; Singh et al., 2019; Warren et al., 2019). Crosstalk exists between extrinsic and intrinsic pathways; BH3-interacting domain death agonist (BID) is cleaved into truncated BID (tBID) by active caspases 8 and 10, translocates to mitochondria, and then interacts with BCL-2 family proteins, which leads to the initiation of apoptosis signaling (Kantari and Walczak, 2011; Kaufmann et al., 2012).

Mitochondrial permeability transition (MPT) promotes the release of <1,500-Da solutes from the matrix *via* the mitochondrial permeability transition pore (MPTP), which ultimately activates cellular responses ranging from mitophagy to cell death (Bonora et al., 2022). Voltage-dependent anion channel (VDAC), adenine nucleotide translocase (ANT), mitochondrial phosphate carrier, and F_1_F_0_-ATPase were initially identified as components of MPTP (Kwong and Molkentin, 2015; Javadov et al., 2017). ANT proteins have been reported to regulate MPTP, but are not essential (Kokoszka et al., 2004; Urbani et al., 2019). VDAC proteins are also dispensable for MPTP (Krauskopf et al., 2006;; Baines et al., 2007; Urbani et al., 2019). Although cyclophilin D is not a component of MPTP, it is a critical modulator of MPTP, desensitizing them to stress (Kwong and Molkentin, 2015; Javadov et al., 2017). VDAC proteins form major channels across MOM, allowing the free passage of small solutes (Maurya and Mahalakshmi, 2016; Mazure, 2017; Magrí et al., 2018). ANT proteins localize to mitochondrial inner membranes (MIM), and mediate the exchange activity of ADP and ATP (Zhivotovsky et al., 2009; Brenner et al., 2011). ANT proteins were recently shown to mediate mitophagy (Hoshino et al., 2019). Therefore, MOMP and MPT regulate mitochondrial stress responses, including cell death and mitophagy.

### Regulation of mitochondrial fragmentation

Mitochondrial fission and fusion processes play an essential role in the compartmentalization of damaged and healthy mitochondria, and are linked to mitophagy (Youle and van der Bliek, 2012). The large GTPase dynamin-related protein 1 (DRP1) mediates mitochondrial fission (Otera et al., 2013; Kraus and Ryan, 2017). Mitochondrial fission 1 (FIS1), mitochondrial fission factor (MFF), and mitochondrial dynamics proteins of 49 and 51 kDa (MiD49 and MiD51) are mitochondrial receptor proteins anchored to MOM and mediate mitochondrial fragmentation (Schrader et al., 2012; Rovira-Llopis et al., 2017). DRP1 is translocated from the cytosol to MOM by binding to mitochondrial receptor proteins, and promotes mitochondrial fission through GTPase activity (Otera et al., 2013; Kraus and Ryan, 2017). In contrast, mitofusins 1 and 2 are GTPases involved in the fusion of MOM, while OPA1 mitochondrial dynamin like GTPase mediates the fusion of MIM (Schrepfer and Scorrano, 2016; Tilokani et al., 2018). During fission and fusion cycles, healthy daughter mitochondria undergo the fusion process, while damaged daughter mitochondria are destined for degradation by mitophagy (Youle and van der Bliek, 2012). The dysfunction of mitochondrial dynamics is associated with cardiovascular diseases and neurodegenerative disorders (Burté et al., 2015; Vásquez-Trincado et al., 2016; Murphy and Hartley, 2018).

### Regulation of mitophagy

Mitophagy plays an essential role in mitochondrial quality control and cellular homeostasis (Ashrafi and Schwartz, 2013; Palikaras et al., 2018; Pickles et al., 2018). When mitochondria are damaged in response to various stresses, they are enclosed by the elongation of phagophores, which leads to the formation of autophagosomes (Lahiri et al., 2019). Autophagosomes then fuse with lysosomes and their contents are destined for degradation (Lahiri et al., 2019). Autophagy-related protein 8 (ATG8) family proteins are required for the formation of autophagosomes, and are divided into microtubule-associated protein 1 light chain 3 (MAP1LC3) and GABA type A receptor-associated protein (GABARAP) family proteins in multicellular animals, which consist of MAP1LC3A, MAP1LC3B, MAP1LC3C, GABARAP, GABARAPL1, and GABARAPL2 (Schaaf et al., 2016; Johansen and Lamark, 2020). Upon the activation of mitophagy by a number of stimuli, MAP1LC3 family proteins are linked to phosphatidylethanolamine and anchored to phagophore membranes, which is an essential step in the formation of autophagosomes (Schaaf et al., 2016; Johansen and Lamark, 2020).

**FIGURE 2:**
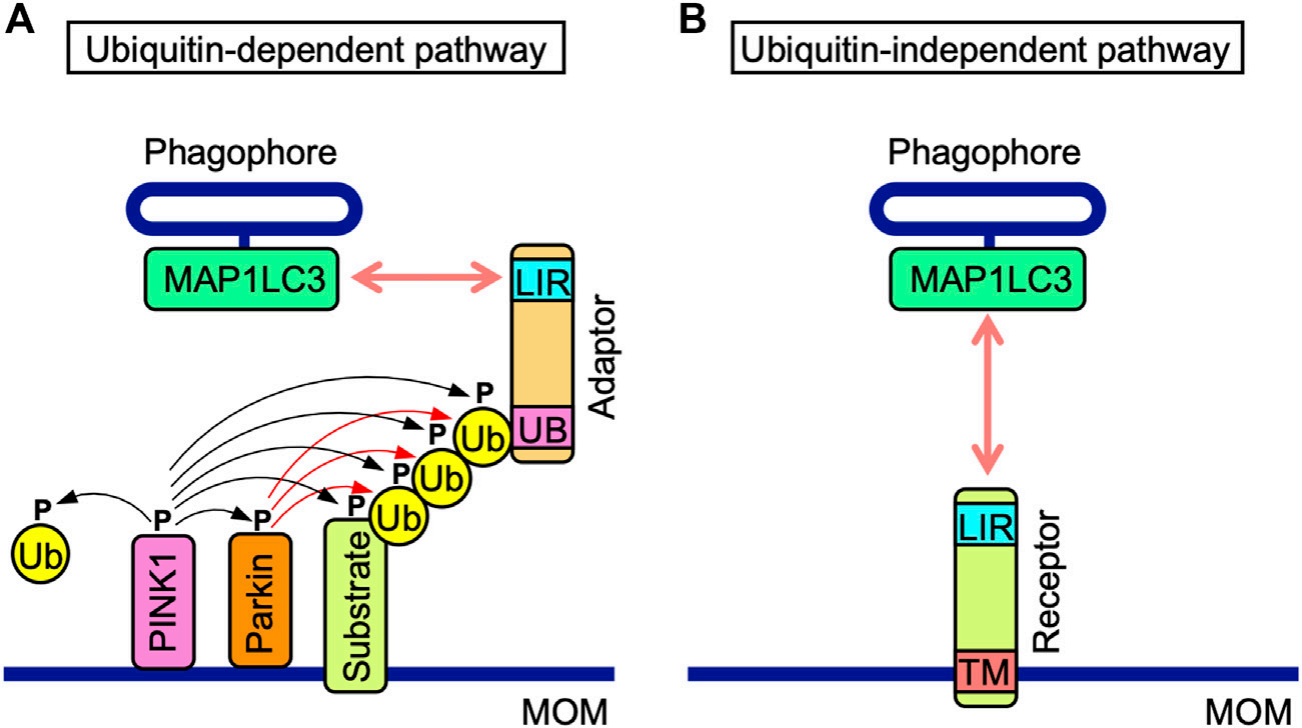
Ubiquitin-dependent and-independent mitophagy pathways. **(A)** In the ubiquitin-dependent pathway, PINK1 is stabilized on mitochondrial outer membranes (MOM) and phosphorylates PINK1 itself, the E3 ubiquitin (Ub) ligase Parkin, MOM-associated substrates, and free and polymerized Ub (black arrows). Parkin ubiquitinates MOM-associated substrates (red arrows). Poly-Ub chains of MOM-associated substrates interact with the ubiquitin-binding domain (UB) of adaptor proteins, which also possess the MAP1LC3-interacting region (LIR) motif. The LIR motif interacts with MAP1LC3 proteins anchored to phagophores. **(B)** Mitophagy receptors harbor the LIR motif and transmembrane (TM) domain anchored to MOM. In the ubiquitin-independent pathway, mitophagy receptors interact with MAP1LC3 proteins anchored to phagophores.

Ubiquitin-dependent and-independent mitophagy both mediate the recruitment of MAP1LC3-associated phagophores to MOM. Ubiquitin-dependent mitophagy is regulated by PINK1 and Parkin (Figure 2A) (Pickrell and Youle, 2015; McWilliams and Muqit, 2017). PINK1 stability is regulated by mitochondrial membrane depolarization (Pickrell and Youle, 2015; McWilliams and Muqit, 2017). PINK1 is stabilized on MOM and phosphorylates various proteins, such as Parkin, MOM substrates, and ubiquitin, while Parkin ubiquitinates MOM proteins (Pickrell and Youle, 2015; McWilliams and Muqit, 2017). Their polyubiquitin chains are phosphorylated by PINK1 and recruit adaptor proteins, which interact with MAP1LC3 family proteins *via* their MAP1LC3-interacting region (LIR) motifs comprising the core consensus sequence of [W/F/Y] XX [L/I/V] (where X is any amino acid) (Birgisdottir et al., 2013). NDP52, SQSTM1, NBR1, TAXBP1, and TOLLIP are adaptor proteins that promote the recruitment of phagophores (McWilliams and Muqit, 2017; De et al., 2021).

In addition to Parkin, ubiquitin-dependent mitophagy is mediated by other E3 ubiquitin ligases, such as mitochondrial E3 ubiquitin protein ligase 1 (MUL1), siah E3 ubiquitin protein ligase 1 (SIAH1), and ariadne RBR E3 ubiquitin protein ligase 1 (ARIH1) (Terešak et al., 2022). MUL1 is anchored to MOM and synergistically promotes mitophagy with PINK1 (Calle et al., 2022). SIAH1 forms a complex with PINK1 and synphilin-1, and mediates mitophagy that does not require Parkin activity (Szargel et al., 2016). ARIH1 triggers PINK1-dependent mitophagy in cancer cells in a Parkin-independent manner (Villa et al., 2017). Therefore, multiple E3 ubiquitin ligases, other than Parkin, mediate PINK1-dependent mitophagy.

The ubiquitin-independent pathway is mediated by MOM-anchored mitophagy receptors, all of which possess LIR motifs (Figure 2B). MOM-anchored mitophagy receptors consist of at least five proteins, i.e., BCL-2/adenovirus E1B 19 kDa protein-interacting protein 3 (BNIP3), BCL-2/adenovirus E1B 19 kDa protein-interacting protein 3-like (BNIP3L), also known as Nip3-like protein X (NIX), FUN14 domain-containing 1 (FUNDC1), FK506-bining protein prolyl isomerase 8 (FKBP8), and BCL-RAMBO. The direct interactions of mitophagy receptors with MAP1LC3 family proteins are a prerequisite for the recognition of mitochondria by autophagosomes.

In addition to MOM-anchored mitophagy receptors, prohibitin 2 (PHB2), cardiolipin, and Autophagy/bectin-1 regulator 1 (AMBRA1) mediate the ubiquitin-independent pathway. PHB2 localizes to MIM and mediates Parkin-induced mitophagy by binding to MAP1LC3 proteins upon mitochondrial depolarization and MOM rupture (Wei et al., 2017). Cardiolipin is a phospholipid that localizes to MIM and acts as a receptor for MAP1LC3 proteins when translocated from MIM to MOM in response to stress (Dudek, 2017; Paradies et al., 2019). AMBRA1 is a mitophagy receptor possessing a LIR motif and mediates Parkin-dependent and -independent mitophagy when it localizes to mitochondria (Strappazzon et al., 2011; Strappazzon et al., 2015). HECT, UBA, and WWE domain containing 1 (HUWE1) belongs to the HECT E3 ubiquitin ligase family and possesses the BH3 domain (Kao et al., 2018). HUWE1 interacts with MCL1 and promotes AMBRA1-dependent mitophagy by mediating MCL1 degradation (Strappazzon et al., 2020). The E3 ubiquitin ligase membrane-associated ring-CH-type finger 5 (MARCHF5) mediates the ubiquitination of FUNDC1 at Lys119 for degradation, and MARCHF5 finely regulates hypoxia-induced mitophagy (Chen et al., 2017). Therefore, ubiquitin-dependent protein degradation by E3 ubiquitin ligases plays a regulatory role in the initiation of mitophagy.

### Structure of BCL-RAMBO

We initially identified BCL-RAMBO as a unique member of the BCL-2 family that induces apoptosis through its overexpression (Kataoka et al., 2001). Approximately 15 years later, BCL-RAMBO was shown to regulate mitophagy and mitochondrial fragmentation (Murakawa et al., 2015). Research conducted in the last 2 decades has revealed that BCL-RAMBO regulates various cellular processes, including apoptosis, mitochondrial fragmentation, and mitophagy.

**FIGURE 3:**
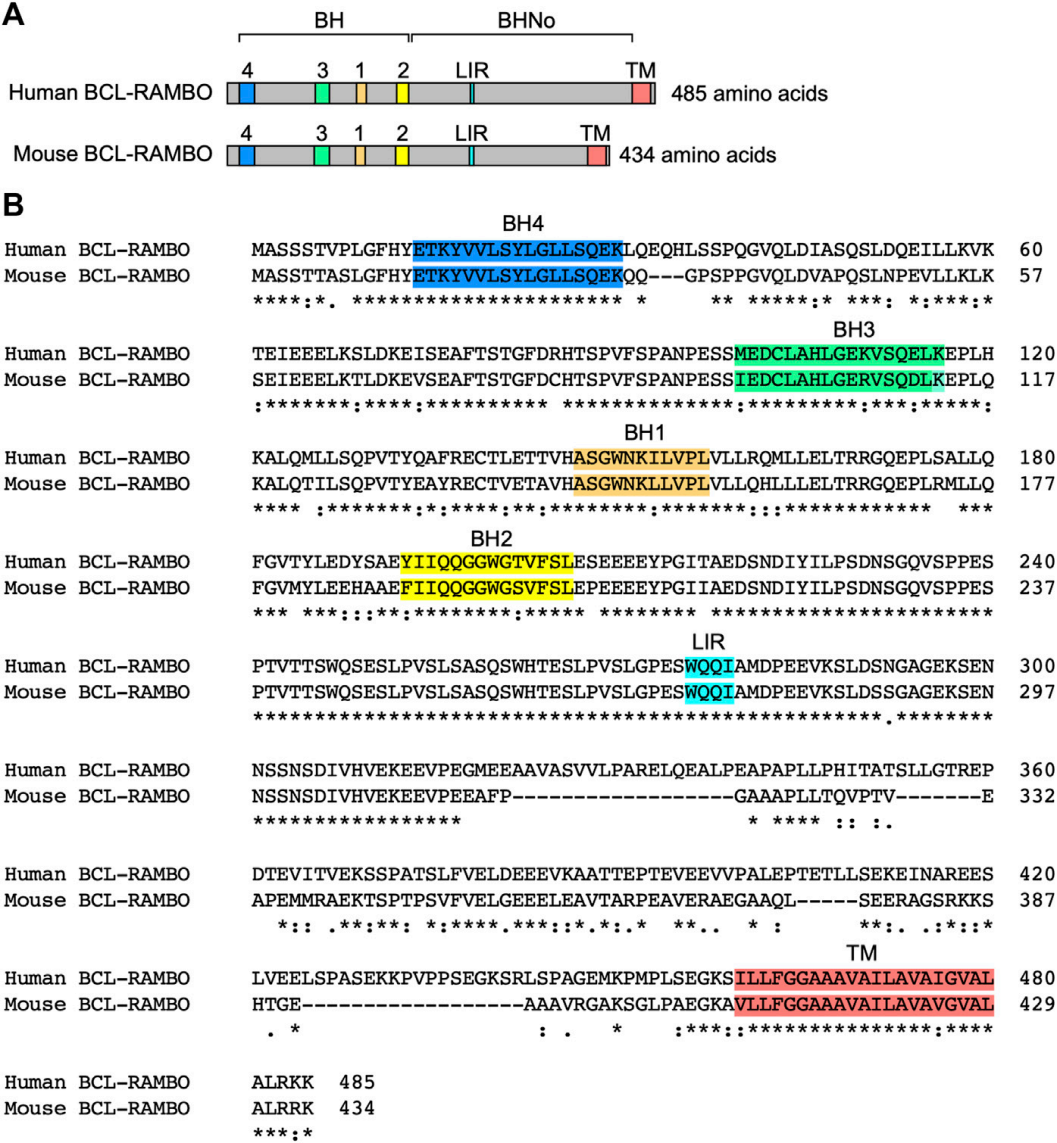
Structures of human and mouse BCL-RAMBO. **(A)** Human BCL-RAMBO consists of 485 amino acids, while mouse BCL-RAMBO consists of 434 amino acids. The BCL-2 homology (BH) domain, No BH motif (BHNo) domain, MAP1LC3-interacting region (LIR) motif, and transmembrane (TM) domain are shown. **(B)** The amino acid sequences of human BCL-RAMBO and mouse BCL-RAMBO are aligned using Clustal Omega (https://www.ebi.ac.uk/Tools/msa/clustalo/).

BCL-2 family proteins are characterized by one to four BH motifs (Adams and Cory 2018; Singh et al., 2019; Warren et al., 2019). Human BCL-RAMBO was initially identified by the screening of public databases for homology with BCL-2 family proteins (Kataoka et al., 2001). Human BCL-RAMBO consists of 485 amino acids and is composed of four N-terminal BH motifs (BH1, BH2, BH3, and BH4), a No BH motif (BHNo) domain, C-terminal transmembrane (TM) domain, and short cytoplasmic tail (Figures 3A, B). Mouse BCL-RAMBO consists of 434 amino acids, which are highly conserved with human BCL-RAMBO, except for the BHNo domain being approximately 50 amino acids shorter than that of human BCL-RAMBO (Figures 3A, B). The BHNo domain is enriched with glutamic acid and serine. Although the predicted molecular mass of human BCL-RAMBO is approximately 55 kDa, the apparent molecular mass of BCL-RAMBO on SDS-PAGE is approximately 85 kDa, which is attributed to the BHNo domain (Kataoka et al., 2001; Yang et al., 2002). In the BHNo domain, 276–279 amino acids of human BCL-RAMBO (WQQI) and 273–276 amino acids of mouse BCL-RAMBO (WQQI) are LIR motifs required for mitophagy (Murakawa et al., 2015). It currently remains unclear whether the difference in the BHNo domain between human BCL-RAMBO and mouse BCL-RAMBO manifests different functions.

### Comparison between BCL-RAMBO and MOM-anchored mitophagy receptors

**FIGURE 4:**
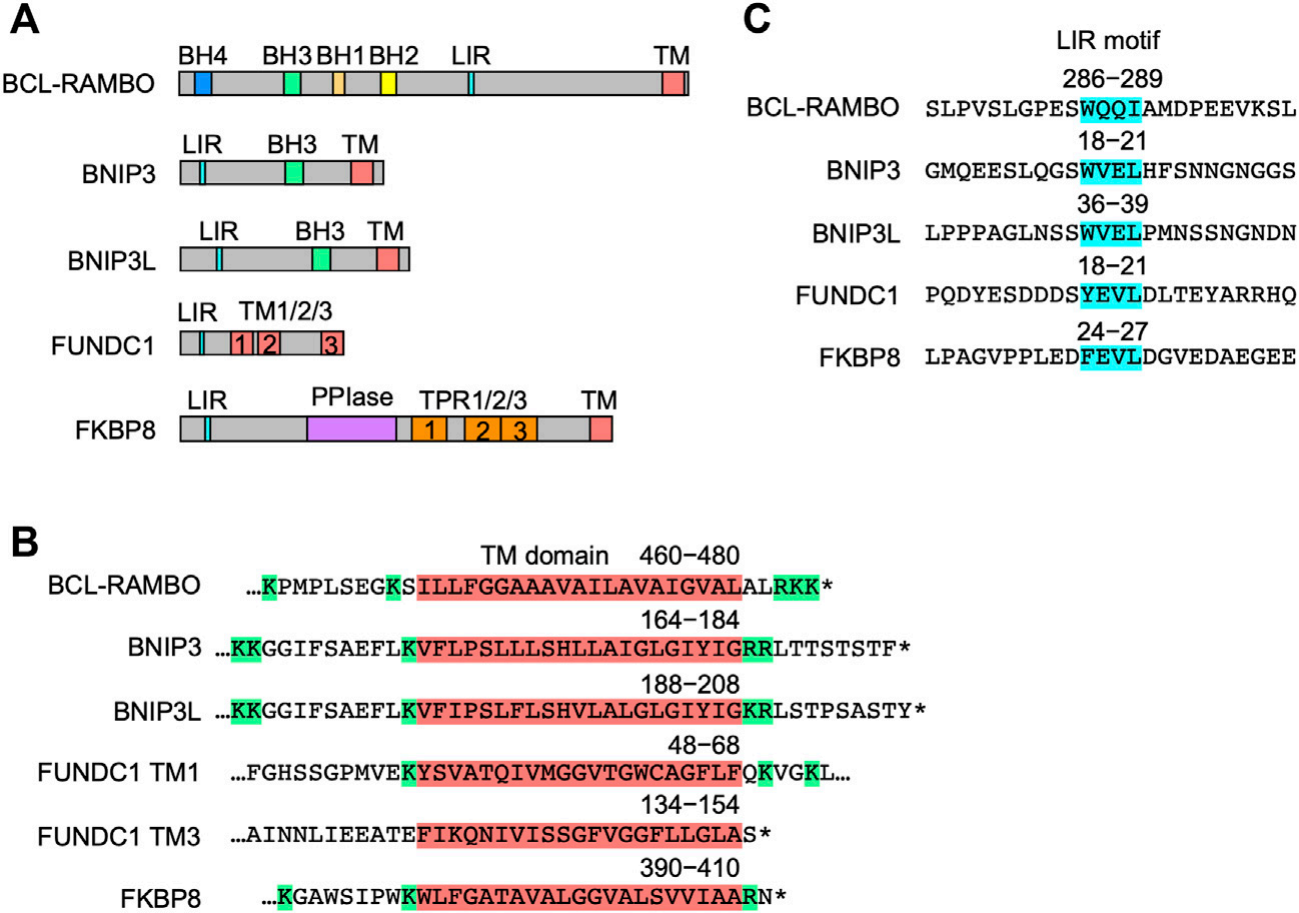
Structures of mitophagy receptors anchored to mitochondrial outer membranes (MOM). **(A)** Mitophagy receptors possess the MAP1LC3-interacting region (LIR) motif and transmembrane (TM) domain. Abbreviations: BCL-2 homology (BH); peptidyl prolyl *cis-trans* isomerase (PPI); tetratricopeptide repeats (TPR). **(B)** Structures of the TM domain of human mitophagy receptors. The TM domain (magenta) is shown with numbers referring to positions in the amino acid sequence. Basic amino acids are shown in green. Asterisks indicate the stop codon. **(C)** Structures of the LIR motif. The LIR motifs (blue) of human mitophagy receptors are shown with numbers referring to positions in the amino acid sequence.

The structures of five MOM-anchored mitophagy receptors, i.e., BNIP3, BNIP3L, FUNDC1, FKBP8, and BCL-RAMBO, are shown in Figure 4A (Otsu et al., 2015; Hamacher-Brady and Brady, 2016; Tan et al., 2016; Yamaguchi et al., 2016; Rodger et al., 2018; Yoo and Jung, 2018; Zhang et al., 2018; Gustafsson and Dorn, 2019; Ma et al., 2020a; Xie et al., 2020; Xu et al., 2020; Meng et al., 2021a; De et al., 2021; Onishi et al., 2021; Onishi and Okamoto, 2021; Poole and Macleod, 2021). In addition to the LIR motif, BCL-RAMBO, BNIP3, BNIP3L, FUNDC1, and FKBP8 commonly possess a C-terminal TM domain followed by a short cytoplasmic tail (1–11 amino acids) (Figure 4B). The C-terminal TM domain flanked at both ends by at least two basic amino acids is required for the mitochondrial targeting of BCL-xL (Kaufmann et al., 2003). Human and mouse BCL-RAMBO both possess a single C-terminal TM domain flanked by two basic amino acids and three basic amino acids (Figure 3B, Figure 4B). The LIR motifs of these mitophagy receptors are present at regions close to the N terminus, except for BCL-RAMBO (Figure 4C). Other regions in the amino acid sequences of mitophagy receptors are not well conserved.

BNIP3 and BNIP3L possess a BH3 domain and are pro-apoptotic proteins (Zhang and Ney, 2009). BCL-2 and BCL-xL interact with BNIP3 and BNIP3L, thereby inhibiting their apoptotic activities (Zhang and Ney, 2009). AMBRA1 is a mitophagy receptor possessing a BH3 domain (Strappazzon et al., 2011; Strappazzon et al., 2015). The C-terminal part of AMBRA1 interacts with BCL-2 and acts as a pro-apoptotic protein upstream of MOMP (Strappazzon et al., 2016). BCL-2 interacts with AMBRA1 and inhibits AMBRA1-induced mitophagy (Strappazzon et al., 2011; Strappazzon et al., 2015). HUWE1 possesses the BH3 domain and mediates MCL1 degradation, while MCL1 delays AMBRA1-dependent mitophagy (Strappazzon et al., 2020). In contrast to these mitophagy receptors harboring the BH3 domain, BCL-RAMBO has not interacted with any of the anti- or pro-apoptotic BCL-2 family proteins examined to date.

### Subcellular localization of BCL-RAMBO

BCL-2 family proteins normally reside in or translocate to mitochondria upon apoptotic stimuli. In addition to mitochondria, BCL-2 family proteins manifest different subcellular localizations to the cytosol, endoplasmic reticulum (ER), nuclear outer membrane, nucleus, Golgi apparatus, and peroxisomes (Popgeorgiev et al., 2018). Previous immunostaining studies showed that endogenous BCL-RAMBO co-localizes with CYCS in human glioma LN235 cells (Jensen et al., 2014) and mitochondrial HSP70 in day 3 human embryos (Boumela et al., 2014). The mitochondrial localization of transfected BCL-RAMBO has been revealed by confocal microscopy (Kataoka et al., 2001; Yi et al., 2003; Kim et al., 2012; Murakawa et al., 2015; Nakazawa et al., 2016) and transmission electron microscopy (Jensen et al., 2014). The C-terminal TM domain of BCL-RAMBO is essential for its mitochondrial localization because BCL-RAMBO lacking the TM domain does not co-localize with mitochondrial marker proteins (e.g. SMAC, HSP60, and ATP synthase) or MitoTracker Red in various cell lines (Kataoka et al., 2001; Yi et al., 2003; Kim et al., 2012; Murakawa et al., 2015; Nakazawa et al., 2016). A mitochondrial fractionation study identified BCL-RAMBO as an integral outer membrane protein with N-terminal BH and BHNo domains exposed to the cytosol and a C-terminal TM domain anchored to MOM (Murakawa et al., 2015). Therefore, BCL-RAMBO mainly localizes to MOM with BH and BHNo domains in the cytosol and a C-terminal short tail in the intermembrane space.

### BCL-RAMBO-binding proteins

**FIGURE 5:**
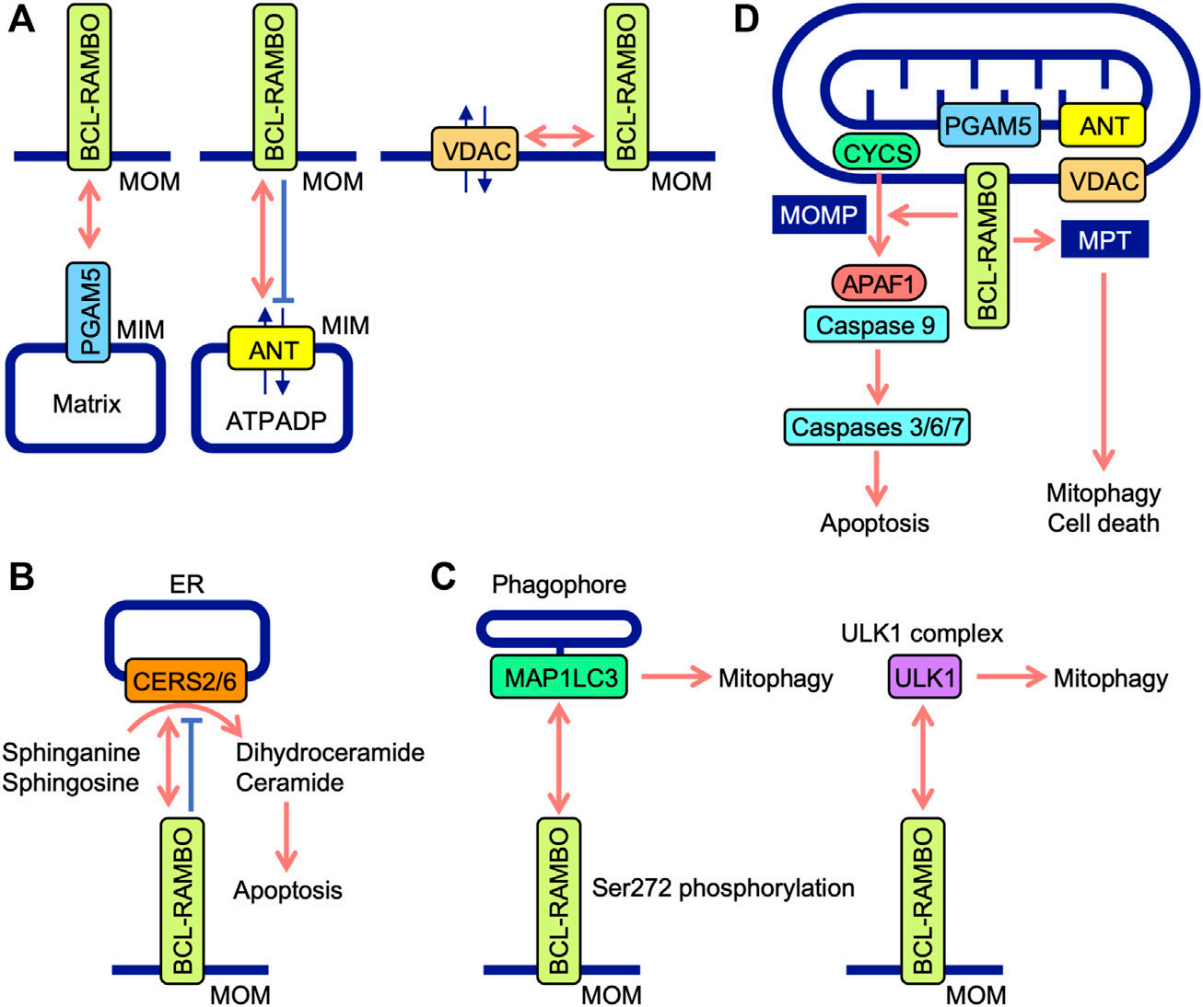
Interaction between BCL-RAMBO and BCL-RAMBO-binding proteins. **(A)** BCL-RAMBO is expressed on mitochondrial outer membranes (MOM). BCL-RAMBO interacts with PGAM5 expressed on the mitochondrial inner membranes (MIM), which is dependent on a transmembrane domain of BCL-RAMBO, but not its C-terminal intermembrane tail. BCL-RAMBO interacts with ANT expressed on the MIM and inhibits ANT activity, which exchanges ATP and ADP. BCL-RAMBO interacts with VDAC expressed on MOM. **(B)** BCL-RAMBO interacts with CERS2 and CERS6, and inhibits their catalytic activities, thereby inhibiting apoptosis induced by dihydroceramide and ceramide. **(C)** BCL-RAMBO interacts with MAP1LC3 proteins *via* its LIR motif. The phosphorylation of BCL-RAMBO at Ser272 is necessary for the interaction with MAP1LC3. BCL-RAMBO interacts with the ULK1 complex, which promotes mitophagy. **(D)** The BCL-RAMBO-dependent activation of caspases is promoted by PGAM5, ANT, and VDAC. BCL-RAMBO induces the mitochondrial release of CYCS *via* mitochondrial outer membrane permeability (MOMP). CYCS initiates the APAF1-dependent activation of caspase-9, and subsequent activation of caspases 3, 6, and 7, leading to apoptosis. BCL-RAMBO induces mitochondrial permeability transition (MPT), which causes mitophagy and cell death.

BH domains mediate the interactions of BCL-2 family proteins (Adams and Cory, 2018; Singh et al., 2019; Warren et al., 2019). Immunoprecipitation assays showed that BCL-RAMBO does not interact with seven anti-apoptotic proteins (e.g., BCL-2, BCL-xL, and MCL1) or six pro-apoptotic proteins (e.g., BAX, BAK, and BIM), but possesses four conserved BH motifs (Kataoka et al., 2001). *Drosophila* possesses two BCL-2 family proteins (Drob-1 and Buffy) (Clavier et al., 2016). Furthermore, BCL-RAMBO does not co-immunoprecipitate with Drob-1 or Buffy (Nakazawa et al., 2016). These findings indicate that BCL-RAMBO does not bind to many BCL-2 family proteins. BCL-RAMBO has so far been shown to bind to ANT, ceramide synthases (CERSs), MAP1LC3/GABARAP, VDAC, unc51-like autophagy-activating kinase 1 (ULK1), phosphoglycerate mutase family member 5 (PGAM5), and SidF (Table 1). These interactions between BCL-RAMBO and its partners appear to regulate apoptosis and mitophagy in mammalian cells (Figures 5A–D).

**TABLE 1:** Biological properties of BCL-RAMBO-binding proteins.

Proteins	Biological properties	References
ANT	BCL-RAMBO binds ANT1	Kim et al. (2012)
	BCL-RAMBO inhibits ADP/ATP translocation activity	
	BCL-RAMBO binds ANT1 and ANT2	Matsubara et al. (2019)
CERS	BCL-RAMBO binds CERS2 and CERS6	Jensen et al. (2014)
	BCL-RAMBO inhibits ceramide synthase activity	
MAP1LC3	BCL-RAMBO binds MAP1LC3B	Murakawa et al. (2015)
	BCL-RAMBO promotes MAP1LC3B-II-mediated mitophagy	
	BCL-RAMBO binds MAP1LC3C, GABARAP, and GABARAPL1	Li et al. (2020)
ULK1	BCL-RAMBO binds ULK1	Murakawa et al. (2019)
VDAC	BCL-RAMBO binds VDAC1	Matsubara et al. (2019)
	VDAC promotes BCL-RAMBO-induced caspase activity	
PGAM5	BCL-RAMBO binds PGAM5	Hashino et al. (2022)
	PGAM5 promotes BCL-RAMBO-induced caspase activity	
	PGAM5 inhibits BCL-RAMBO-induced mitophagy	
SidF	SidF binds BCL-RAMBO and BNIP3	Banga et al. (2007)
	SidF inhibits BCL-RAMBO-induced apoptosis	

### Regulation of apoptosis by BCL-RAMBO

The overexpression of BCL-RAMBO induces apoptosis in the human cancer cell lines, 293T (Kataoka et al., 2001), MCF-7 (Banga et al., 2007), and PC-3 (Kim et al., 2012). BCL-RAMBO promotes taxol-induced cell death in 293A cells and etoposide-induced cell death in HeLa cells (Yi et al., 2003). Wild-type BCL-RAMBO and BCL-RAMBO lacking the N-terminal BH domain, but not BCL-RAMBO lacking the C-terminal TM domain, similarly induces caspase activity by their overexpression in 293T cells (Kataoka et al., 2001). The overexpression of BCL-RAMBO, but not BCL-RAMBO lacking the TM domain, induces apoptosis in PC-3 cells (Kim et al., 2012). These findings indicate that the BHNo domain, but not the BH domain, is required for caspase activation by BCL-RAMBO. This is consistent with BCL-RAMBO not interacting with many anti-apoptotic BCL-2 family members or pro-apoptotic BCL-2 family members (Kataoka et al., 2001). BCL-RAMBO-induced caspase activation occurs independently of death receptor signaling, and, thus, is insensitive to a dominant-negative version of the adaptor protein FADD or the caspase 8 modulator c-FLIP_L_ (Kataoka et al., 2001). Therefore, the BHNo domain of BCL-RAMBO is indispensable for inducing apoptosis signaling in mitochondria.

Previous study demonstrated that BCL-RAMBO induces the release of CYCS from mitochondria, which is not prevented by the caspase inhibitor Z-VAD-fluoromethylketone (Kataoka et al., 2001). Caspase activity induced by BCL-RAMBO is inhibited by IAP proteins (i.e., cIAP1, cIAP2, and XIAP) (Kataoka et al., 2001). These findings suggest that BCL-RAMBO induces MOMP to release CYCS, which triggers the APAF1-dependent activation of caspase 9, leading to caspases 3, 6, and 7 activation and the execution of apoptosis (Figure 5D).

The ANT family of proteins localize to mitochondria, mediate translocation, and exchange ATP and ADP (Zhivotovsky et al., 2009; Brenner et al., 2011). Four ANT isoforms (ANT1, ANT2, ANT3, and ANT4) have different tissue distributions (Brenner et al., 2011). ANT1 and ANT3 are suggested to be pro-apoptotic, while ANT2 and ANT4 are anti-apoptotic (Brenner et al., 2011). These findings indicate that ANT proteins are involved in the regulation of apoptosis. BCL-RAMBO, but not BCL-RAMBO lacking a TM domain, interacts with ANT1 and inhibits ADP/ATP translocation activity in isolated mitochondria (Kim et al., 2012). The knockdown of ANT1 and ANT2 by siRNAs reduces cell death induced by the overexpression of BCL-RAMBO in PC-3 cells (Kim et al., 2012). Consistent with this finding, we showed that BCL-RAMBO physically interacts with ANT1 and ANT2 (Matsubara et al., 2019). BCL-RAMBO induces MPT in isolated mitochondria, which is prevented by bongkrekic acid (Kim et al., 2012). Bongkrekic acid inhibits ANT activity rather than the electron transport chain (Anwar et al., 2017). In addition to MOMP, BCL-RAMBO has been proposed to induce MPT in a manner that is dependent on ANT proteins, leading to the initiation of mitophagy and cell death (Figure 5D).

VDAC proteins, also known as mitochondrial porin, form major channels for small metabolites at MOM, and consist of three isoforms, VDAC1, VDAC2, and VDAC3, in mammals (Mazure, 2017; Magrí et al., 2018). VDAC1 mainly exhibits pro-apoptotic activity, while VDAC2 shows anti-apoptotic activity (Maurya and Mahalakshmi, 2016; Magrí et al., 2018). In *Drosophila*, the gene porin is widely expressed in adult tissues, while porin*2* is exclusively expressed in spermatozoa (Graham and Craigen, 2005; Guarino et al., 2006). *Drosophila* porin affects the morphology of mitochondria (Park et al., 2010). Comprehensive genetic screening using *Drosophila* fly lines deficient for the second chromosome showed that human BCL-RAMBO genetically interacts with porin (Matsubara et al., 2019). Glutathione S-transferase-tagged human BCL-RAMBO interacts with VDAC1, albeit to a lesser extent than ANT2 (Matsubara et al., 2019). The co-transfection of VDAC1 or VDAC2 with BCL-RAMBO promotes the cleavage of procaspase 7 and DEVD-hydrolyzing activity in HEK293T cells (Matsubara et al., 2019). These findings indicate that BCL-RAMBO and VDAC cooperatively promote the activation of effector caspases in mammalian cell lines.

Human CERSs comprise six isoforms and catalyze the acylation of sphinganine and sphingosine to convert dihydroceramide and ceramide, respectively (Pewzner-Jung et al., 2006; Wegner et al., 2016). CERS activity mainly localizes to the cytoplasmic leaflet of the ER (Pewzner-Jung et al., 2006). Ceramides are necessary for MOMP and CYCS release (Ueda, 2015). Yeast two-hybrid screening revealed that CERS2 is an interacting partner of BCL-RAMBO (Jensen et al., 2014). BCL-RAMBO binds to CERS2 and CERS6 *via* the BHNo domain and inhibits their homo- and hetero-dimerization and enzyme activity (Jensen et al., 2014). The overexpression of BCL-RAMBO decreases staurosporine-induced CYCS release, caspase activation, and apoptosis, while its knockdown enhances staurosporine-induced caspase activation and apoptosis (Jensen et al., 2014). Based on these findings, BCL-RAMBO has been proposed to inhibit ceramide-dependent apoptosis by preventing CERS2 and CERS6 and exhibiting anti-apoptotic activity (Figure 5B). The progression of glioblastoma is impaired by the depletion of BCL-RAMBO and enhanced by its overexpression (Jensen et al., 2014). In mouse 3T3-L1 cells cultured under adipogenic and non-differentiation conditions, the knockdown of BCL-RAMBO decreases apoptosis (Fujiwara et al., 2019). In addition to pro-apoptotic activities, BCL-RAMBO exhibits anti-apoptotic activity. Collectively, these findings indicate that BCL-RAMBO exhibits both pro- and anti-apoptotic activities in cellular context-dependent manners.

### Regulation of mitophagy by BCL-RAMBO

The screening of the Uniprot database identified BCL-RAMBO as a mammalian Atg32 homologue that shares the WXXI motif, acidic amino acid clusters, and a single TM domain for mitochondrial localization (Murakawa et al., 2015). A yeast two-hybrid assay and pulldown assay revealed that BCL-RAMBO binds to MAP1LC3B *via* the LIR motif (Murakawa et al., 2015). BCL-RAMBO augments the expression of MAP1LC3B-II, a lipidated form of MAP1LC3B required for the recruitment of phagophores, and promotes mitophagy in a Parkin-independent manner (Murakawa et al., 2015). Carbonyl cyanide 3-chlorophenylhydrazone (CCCP)-induced mitophagy is attenuated by the knockdown of BCL-RAMBO in HEK293A cells (Murakawa et al., 2015). In the *Drosophila* genetic complementation assay, Atg8a genetically interacts with human BCL-RAMBO (Nakazawa et al., 2016). A subsequent study showed the more selective binding of BCL-RAMBO to MAP1LC3C, GABARAP, and GABARAPL1 than to other MAP1LC3/GABARAP family proteins (Li et al., 2020). Upon transfection in 293A and 293T cells, BCL-RAMBO increases MAP1LC3B-II levels (Murakawa et al., 2015; Hashino et al., 2022). These findings indicate that BCL-RAMBO promotes mitophagy *via* interactions with MAP1LC3 family proteins. The Ser272 residue of mouse BCL-RAMBO is phosphorylated by its overexpression, while S272A mutants show a reduction in MAP1LC3 binding and MAP1LC3B-II protein levels (Murakawa et al., 2015). BCL-RAMBO is currently proposed to be phosphorylated at Ser272 proximal to the LIR motif and promotes the interaction with MAP1LC3, recruiting phagophores (Figure 5C).

The ULK1 complex consists of ULK1, FAK family kinase-interacting of 200 kDa, ATG13, and ATG101 and initiates the formation of autophagosomes (Lin and Hurley, 2016; Zachari and Ganley, 2017). A recent study showed that BCL-RAMBO co-immunoprecipitates with endogenous ULK1 when transfected in 293 A cells (Murakawa et al., 2019). Endogenous BCL-RAMBO also immunoprecipitates with endogenous ULK1 in CCCP-treated cells (Murakawa et al., 2019). Therefore, BCL-RAMBO appears to recruit the ULK1 complex to MOM for the formation of autophagosomes. In yeast, the interaction of BCL-RAMBO with Atg1 and Atg8 is important for the initiation of BCL-RAMBO-mediated mitophagy (Murakawa et al., 2019). Collectively, these findings demonstrate that Atg8/MAP1LC3 and Atg1/ULK1 proteins are necessary for BCL-RAMBO-mediated mitophagy. BCL-RAMBO interacts with the ULK1 complex and promotes mitophagy (Figure 5C).

Mitophagy receptors anchored to MOM are regulated by phosphorylation and dephosphorylation (Table 2). Binding to MAP1LC3/GABARAP family proteins is promoted by the phosphorylation of serine residues adjacent to the LIR motif at Ser272 in BCL-RAMBO, Ser17 and Ser24 in BNIP3, Ser34 and Ser35 in BNIP3L, and Ser17 in FUNDC1 (Zhu et al., 2013; Wu et al., 2014b; Murakawa et al., 2015; Rogov et al., 2017), whereas FKBP8 does not possess potential phosphorylated amino acid residues close to the LIR motif (Figure 4C). In contrast, the dephosphorylation of Ser13 and Tyr18 activates FUNDC1-mediated mitophagy in response to hypoxia (Liu et al., 2012; Wu et al., 2014a; Chen et al., 2014). Several protein kinases have been reported to target FUNDC1: casein kinase 2 (CK2) phosphorylates Ser13 (Chen et al., 2014), ULK1 phosphorylates Ser17 (Wu et al., 2014b), and SRC proto-oncogene, non-receptor tyrosine kinase phosphorylates Tyr18 (Liu et al., 2012). Other than MOM-anchored mitophagy receptors, AMBRA1 is phosphorylated by inhibitor of nuclear factor κB kinase subunit α (IKK-α) at Ser1014, which is proximal to the LIR motif WEQL at 1,020–1,023 amino acids (Di Rita et al., 2018). IKK-α-dependent phosphorylation promotes structural changes in AMBRA1 and promotes its interaction with MAP1LC3 (Di Rita et al., 2018). In contrast, PGAM5 dephosphorylates FUNDC1 at Ser13 (Chen et al., 2014). Therefore, the interactions between mitophagy receptors and MAP1LC3/GABARAP family proteins are regulated by phosphorylation and dephosphorylation at LIR and its adjacent regions.

**TABLE 2:** Phosphorylation and dephosphorylation of mitophagy receptors.

Receptors	Positions	Biological activities	References
BCL-RAMBO	Ser272	Ser272 phosphorylation promotes mitophagy	Murakawa et al. (2015)
BNIP3	Ser17	Ser17 and Ser24 phosphorylation promotes binding to MAP1LC3B and GABARAPL2	Zhu et al. (2013)
	Ser24		
BNIP3L	Ser34	Ser34 and Ser35 phosphorylation enhances binding to MAP1LC3B	Rogov et al. (2017)
	Ser35		
	Ser81	Ser81 phosphorylation is critical for ischemia-reperfusion-induced mitophagy	Yuan et al. (2017)
	Ser212	PRKA phosphorylates Ser212	da Silva Rosa et al. (2021)
FUNDC1	Ser13	Casein kinase 2 phosphorylates Ser13	Chen et al. (2014)
		PGAM5 dephosphorylates Ser13	
		Ser13 dephosphorylation enhances binding to MAP1LC3	
		Ser13 dephosphorylation activates hypoxia-induced mitophagy	Wu et al. (2014a)
	Ser17	ULK1 phosphorylates Ser17	Wu et al. (2014b)
		Ser17 phosphorylation enhances binding to MAP1LC3	
	Tyr18	SRC kinase phosphorylates Tyr18	Liu et al. (2012)
		Tyr18 dephosphorylation activates hypoxia-induced mitophagy	

In contrast to mammalian mitophagy receptors, the LIR motif WQAI at 86–89 amino acids of yeast Atg32 does not appear to play a major role in mitophagy (Liu et al., 2014; Furukawa et al., 2019). Ser114 and Ser119 are phosphorylated in yeast Atg32, which mediates the interaction between Atg11 and Atg32 and subsequent mitophagy (Aoki et al., 2011). CK2 phosphorylates Ser114 and Ser119 (Kanki et al., 2013), while the protein phosphatase 2A-like protein phosphatase Ppg1 dephosphorylates Atg32, thereby preventing mitophagy (Furukawa et al., 2018). In the case of BNIP3L, the phosphorylation of Ser81, which is distant from the LIR motif, is essential for BNIP3L-dependent mitophagy (Yuan et al., 2017). Protein kinase cAMP-activated (PRKA) phosphorylates BNIP3L at Ser212 within the C terminus of the TM domain and distal from the LIR motif WVEL at amino acids 36–39 (da Silva Rosa et al., 2021). Other than MOM-anchored mitophagy receptors, PHB2 is phosphorylated by Aurora kinase A at Ser39 distant from the LIR motif YPII at amino acids 121–124, which promotes the interaction between PHB2 and MAP1LC3 (Bertolin et al., 2021). In addition to the LIR motif and its surroundings, mammalian mitophagy receptors are regulated by phosphorylation at other regions distant from the LIR motif to initiate mitophagy.

### Regulation of mitochondrial fragmentation by BCL-RAMBO

The BH domain of BCL-RAMBO is responsible for mitochondrial fragmentation in 293A cells, while DRP1 is not required for BCL-RAMBO-induced mitochondrial fragmentation (Murakawa et al., 2015). The overexpression of the BCL-RAMBO S272A mutant induces mitochondrial fragmentation and also reduces MAP1LC3 binding (Murakawa et al., 2015). We showed that the ectopic expression of BCL-RAMBO induces the fragmentation of mitochondria and their perinuclear accumulation in A549 cells (Matsubara et al., 2019). These findings clearly indicate that BCL-RAMBO induces mitochondrial fragmentation *via* the BH domain, but not the LIR motif. The BH domain of BCL-RAMBO possesses the BH domain consisting of BH1, BH2, BH3, and BH4, in contrast to other mitophagy receptors (BNIP3, BNIP3L, and AMBRA1), which only possess the BH3 domain. Moreover, BCL-RAMBO does not interact with BCL-2 or BCL-xL (Kataoka et al., 2001). Therefore, unlike BNIP3, BNIP3L, and AMBRA1, the BH domain of BCL-RAMBO is unique for the regulation of mitochondrial fragmentation.

Human lung adenocarcinoma A549 cells are more susceptible to the accumulation of fragmented mitochondria by BCL-RAMBO transfection than other cultured cell lines used for subcellular localization (e.g., HeLa, 293A, and PC-3 cells). In A549 cells, the knockdown of three VDAC isoforms (VDAC1, VDAC2, and VDAC3) induces mitochondrial fragmentation, but not their perinuclear accumulation (Matsubara et al., 2019). In *Drosophila*, porin mutations cause elongated mitochondria, while porin overexpression results in fragmented mitochondria (Park et al., 2010). These findings clearly indicate that VDAC proteins are involved in mitochondrial fragmentation; however, the outcome of the knockdown of VDAC in mammalian cell lines differs from that of VDAC mutations in *Drosophila*. In mammalian cells, a reduction in VDAC proteins may lead to mitochondrial dysfunction, thereby promoting mitochondrial fragmentation. The MOM-anchored receptors, FIS1, MFF, MiD49, and MiD51 recruit DRP1 to mitochondrial fission sites (Schrader et al., 2012; Rovira-Llopis et al., 2017). Since BCL-RAMBO mediates DRP1-independent mitochondrial fragmentation (Murakawa et al., 2015), it may also promote mitochondrial fragmentation by regulating FIS1, MFF, MiD49, and MiD51 at a step downstream of DRP1.

### Regulation of BCL-RAMBO-induced apoptosis and mitophagy by PGAM5

PGAM family proteins possess a conserved PGAM domain that catalyzes mutase and/or phosphatase activities (Sadatomi et al., 2013; Cheng et al., 2021; Liang et al., 2021). PGAM5 lacks mutase activity, but mediates phosphatase activity and plays a regulatory role in mitochondrial dynamics and cell death (Sadatomi et al., 2013; Cheng et al., 2021; Liang et al., 2021). PGAM5 localizes to mitochondria *via* its N-terminal TM domain (Lo and Hannink, 2008). In mitochondrial compartments, PGAM5 localizes to MIM (Sekine et al., 2012; Lu et al., 2014).

Phenotypic screening using *Drosophila* deficiency lines identified candidate genes interacting genetically with human BCL-RAMBO. The Pgam5-2 mutant fly line and Pgam 5-2 knockdown fly line suppress the rough eye phenotype induced by BCL-RAMBO (Hashino et al., 2022). Furthermore, PGAM5 modulates the PINK1-mediated pathway in *Drosophila* (Imai et al., 2010). In human 293T cells, BCL-RAMBO binds to PGAM5, and the BCL-RAMBO TM domain is required for their interaction (Hashino et al., 2022). PGAM5 promotes BCL-RAMBO-induced caspase 3/7 activity, but suppresses the increase of MAP1LC3B-II levels induced by BCL-RAMBO (Hashino et al., 2022). These findings reveal that PGAM5 promotes BCL-RAMBO-induced apoptosis and conversely suppresses BCL-RAMBO-induced mitophagy. BCL-RAMBO localizes to MOM and does not appear to dynamically alter subcellular localization. Phosphorylation and dephosphorylation processes are proposed to regulate BCL-RAMBO-induced apoptosis and mitophagy.

PGAM5 regulates mitochondrial processes by interactions with and the dephosphorylation of multiple proteins. PGAM5 dephosphorylates DRP1 at Ser637 and promotes mitochondrial fragmentation and necrosis (Wang et al., 2012). In contrast, PGAM5 confers protection against necroptosis (Lu et al., 2016). It also interacts with BCL-xL (Lo and Hannick, 2006; Wu et al., 2014a). PGAM5 dephosphorylates BCL-xL at Ser62, restores its binding to BAX and BAK, and provides protection against apoptosis in vinblastine-treated cells (Ma et al., 2020b). Furthermore, PGAM5 regulates Keap1-dependent BCL-xL degradation and exerts anti-apoptotic effects in cardiomyocytes (Yang et al., 2017). In contrast, PGAM5 is required for the activation of BAX and DRP1 dephosphorylation in arenobufagin- and staurosporine-induced apoptosis (Xu et al., 2015). PGAM5 interacts with apoptosis inducing factor and triggers mitophagic cell death, which is inhibited by XIAP (Lenhausen et al., 2016). Therefore, PGAM5 promotes or prevents cell death, including apoptosis and necroptosis, by different mechanisms. In addition to cell death, PGAM5 plays a regulatory role in mitophagy. It dephosphorylates FUNDC1 at Ser13, which promotes mitophagy (Chen et al., 2014). It also interacts with FUNDC1, which requires Syntaxin 17 (Sugo et al., 2018). Moreover, PGAM5 regulates PINK1- and Parkin-mediated mitophagy *via* DRP1 in CCCP-treated cells (Park et al., 2018). These findings reveal that PGAM5 promotes FUNDC1-dependent mitophagy and PINK1- and Parkin-dependent mitophagy. Ser272 adjacent to the LIR motif of mouse BCL-RAMBO is phosphorylated, which promotes mitophagy (Murakawa et al., 2015). These findings suggest that PGAM5 dephosphorylates BCL-RAMBO at Ser272 to prevent mitophagy; however, PGAM5 may also prevent BCL-RAMBO-mediated mitophagy *via* other dephosphorylated sites and/or proteins. The protein kinases and protein phosphatases targeting BCL-RAMBO have not yet been identified.

### Regulation of adipogenesis by BCL-RAMBO

Brown and beige adipocytes share similar morphological and biochemical characteristics and control systemic energy homeostasis through their thermogenic activity (Harms and Seale, 2013; Rosen and Spiegeman, 2014; Ikeda et al., 2018). In contrast to classical brown adipocytes, beige adipocytes are induced to emerge within white adipose tissues and harbor uncoupling protein 1 (UCP1) (Chouchani et al., 2019). BCL-RAMBO and UCP1 are both increasingly expressed during beige and white adipocyte differentiation (Ju et al., 2018). In response to cold exposure or the β3 adrenergic agonist CL-316,243, the expression of BCL-RAMBO and UCP1 is up-regulated in inguinal white adipose tissue (Ju et al., 2018). The knockdown of BCL-RAMBO inhibits the expression of several genes that are necessary for the browning program during the differentiation of inguinal white adipose tissue (Ju et al., 2018). Therefore, BCL-RAMBO appears to play a novel role in promoting beige adipocyte biogenesis.

Pluripotent mesenchymal stem cells are present in bone marrow stromal cells (BMSCs) and differentiate into different cell types, such as osteoblasts and adipocytes (Chen et al., 2016). BCL-RAMBO expression in BMSCs is higher in an adipogenic culture than in an osteogenic culture (Fujiwara et al., 2019). The knockdown of BCL-RAMBO inhibits the differentiation of adipocytes, oxidative phosphorylation, and apoptosis in mouse 3T3-L1 cells (Fujiwara et al., 2019). These findings indicate that BCL-RAMBO promotes adipogenesis through the regulation of oxidative phosphorylation and apoptosis.

### Regulation of bacterial infection by BCL-RAMBO

Programmed cell death plays an essential role in the elimination of cells infected with bacterial pathogens, which target many cellular proteins (Böhme and Rudel, 2009; Ashida et al., 2011). SidF, a substrate of the Dot/Icm transporter system in *Legionella pneumophila*, confers resistance to apoptosis in *L. pneumophila*-infected macrophages (Banga et al., 2007). Yeast two-hybrid assays showed that BCL-RAMBO and BNIP3 interact with SidF (Banga et al., 2007). SidF inhibits BCL-RAMBO-induced apoptosis in human breast cancer MCF-7 cells (Banga et al., 2007). In contrast, the *L. pneumophila*-induced cell death of macrophages is independent of SidF and unaffected by a deficiency in BCL-RAMBO and BNIP3 (Speir et al., 2017). Therefore, the role of BCL-RAMBO in bacterial infection remains controversial and warrants further study.

### Expression of BCL-RAMBO in normal cells and cancer cells

Northern blots of normal human tissues showed that human BCL-RAMBO mRNA has multiple splicing forms and is broadly expressed in all tissues tested, while higher expression is observed in the heart, skeletal muscle, and pancreas (Kataoka et al., 2001). Furthermore, human BCL-RAMBO is constitutively expressed during early embryonic development (Boumela et al., 2014). BCL-RAMBO is highly expressed in skeletal muscle (Ávila-Arcos et al., 2019). The content of BCL-RAMBO in skeletal muscle is higher in controls than in sedentary adults, suggesting that chronic exercise maintains BCL-RAMBO levels in skeletal muscle (Arribat et al., 2018). Mitophagy receptors, including BNIP3, BNIP3L, FUNDC1, and BCL-RAMBO, are expressed in adult cardiac progenitor cells, and their expression remains unchanged during differentiation (Lampert et al., 2019). Proteomic profiling showed that BCL-RAMBO is one of the common proteins in human and mouse lens-enriched membrane proteins (Aryal et al., 2020). BCL-RAMBO expression is up-regulated during the browning process of white adipose tissue (Ju et al., 2018) and adipogenic differentiation in mouse ear mesenchymal stem cells and the mouse 3T3-L1 preadipocyte cell line (Fujiwara et al., 2019). Therefore, BCL-RAMBO is widely expressed in adult tissues, and its expression is more abundant in specific tissues, such as skeletal muscle.

BCL-RAMBO protein expression levels differ in human and mouse cancer cell lines (Kataoka et al., 2001). BCL-RAMBO is expressed in various cancers, including childhood acute lymphoblastic leukemia (ALL), gastric cancer, glioblastoma, and acute myeloblastic leukemia (Holleman et al., 2006; Tahir et al., 2010; Yang et al., 2010; Wang et al., 2013; Jensen et al., 2014; Handschuh et al., 2021). In ALL patients, BCL-RAMBO is involved in resistance to L-asparaginase, which is widely used to treat ALL (Holleman et al., 2006). BCL-RAMBO is an independent prognostic factor for ALL (Holleman et al., 2006; Yang et al., 2010) and is associated with a poor clinical outcome for acute myeloid leukemia (Handschuh et al., 2021). In contrast, in a cohort analysis of node-negative breast cancer, BCL-RAMBO expression is associated with a better prognosis (Petry et al., 2010). The BCL-RAMBO transcript is down-regulated in locally advanced rectal cancer that does not respond to chemotherapy (Millino et al., 2017). The down-regulation of BCL-RAMBO is associated with a poor prognosis in clear cell and papillary renal cell carcinoma (Meng et al., 2021b). Collectively, these findings indicate that BCL-RAMBO promotes or prevents the progression of different types of cancers. These opposing roles of BCL-RAMBO are attributed to the different contributions of the pro-apoptotic activity of BCL-RAMBO and its mitophagy-inducing activity counteracting cell death.

### Transcriptional regulation of BCL-RAMBO

Several transcription factors have been shown to regulate the transcription of the BCL-RAMBO gene. The Zinc-finger of the cerebellum gene (ZIC) was discovered as the *Drosophila melanogaster* pair rule gene *odd-paired*, and ZIC proteins consisting of five tandem C2H2-type zinc fingers regulate gene expression by classical transcription factors and co-factors in mammalian cells (Houtmeyers et al., 2013). Four target genes (i.e. *BCL-RAMBO*, *JunD*, *Fam57A*, and *EIF3M*) are down-regulated by the knockdown of ZIC1 in liposarcoma cell lines (Brill et al., 2010). The Forkhead box O (FOXO) transcription factor family of proteins are regulated by the insulin signaling pathway and promote target genes involved in various processes, such as cell death and cell cycle arrest (Brown and Webb, 2018; Yadav et al., 2018). Runt-related transcription factor (RUNX) family proteins play a role in development, such as hematopoiesis (Mevel et al., 2019). In a 3D breast epithelial culture, the expression of BCL-RAMBO correlates with FOXO1 at the single-cell level and is controlled by RUNX1 (Wang et al., 2011). A chromatin immunoprecipitation assay revealed that FOXO and RUNX consensus binding sites are present in the promoter region of the BCL-RAMBO gene (Wang et al., 2011). Additional sex comb-like 1 (ASXL1) is a member of the Polycomb group of proteins and mediates transcriptional repression (Katoh, 2013). BCL-RAMBO expression is up-regulated and accompanied by reduced global levels of H3K27me3 and H3K4me3 in mouse Asxl1 ^−/−^ hematopoietic stem/precursor cells (Wang et al., 2014). BCL-RAMBO has been identified as one of the 179 genes repressed by the overexpression of anti-silencing function 1B, a conserved histone chaperone, in human MIN6 pancreatic islets (Paul et al., 2016). Limited information is currently available on the transcriptional regulation of BCL-RAMBO; however, the transcription factors responsible for the BCL-RAMBO gene are very important for elucidating the physiological and pathological roles of BCL-RAMBO.

### Transcriptional regulation of BCL-RAMBO by miscellaneous experimental conditions

The mRNA expression of BCL-RAMBO has been associated with one of many fluctuating genes by miscellaneous agents. BCL-RAMBO is one of the seven pro-apoptotic genes with expression levels that decrease in human LU-HNSCC-7 squamous cell carcinoma xenografted in nude mice receiving erythropoietin, which is used to treat anemia in cancer patients (Lindgren et al., 2014). These findings indicate that erythropoietin manifests an adverse effect by inhibiting apoptosis in cancer cells. BCL-RAMBO is one of the four apoptotic genes up-regulated by lithocholic acid and bilirubin, which exert deleterious effects on osteoblast functions, in human Saos-2 osteosarcoma cells (Ruiz-Gaspà et al., 2020). BCL-RAMBO is also one of the 13 key genes up-regulated by *Periplaneta americana*-derived small peptides, which confer resistance to hydrogen peroxide-induced apoptosis in human ovarian granular KGN cells (Wang et al., 2022). TaqMan arrays on apoptotic gene expression revealed that BCL-RAMBO, in addition to BCL-2-binding component 3, is expressed at higher levels in euploid embryos than in aneuploid embryos (Lal et al., 2022). A bioinformatics analysis identified BCL-RAMBO as one of the seven top hub genes down-regulated by an influenza A virus infection (Chen et al., 2022), indicating that BCL-RAMBO is involved in influenza A virus-induced mitophagy. Collectively, these findings show that BCL-RAMBO expression fluctuates to control its pro-apoptotic or mitophagy-inducing activity.

Small-molecule compounds or therapeutic drugs have been reported to affect the transcription of BCL-RAMBO. Cardamonin is a natural compound belonging to the flavonoid family and exhibits anti-cancer activity (Nawaz et al., 2020). BCL-RAMBO is one of the 43 genes down-regulated by cardamonin (1.66 to 6.18-fold) in mouse WEHI-3 leukemia cells (Liao et al., 2019). Cardamonin possesses an α,β-unsaturated carbonyl moiety, which reacts with cysteine residues. Cardamonin targets various cellular proteins, including signaling molecules and transcription factors (Nawaz et al., 2020). Due to the diverse effects of cardamonin, the mechanisms underlying the down-regulation of BCL-RAMBO by cardamonin currently remain unclear. The role of BCL-RAMBO expression in retinal diseases has been examined. Ciprofloxacin is one of the fluoroquinolones currently used as bactericidal antibiotics. It up-regulates BCL-RAMBO expression at the transcriptional level, and this is accompanied by an increase in caspases 3 and 9 in human adult retinal pigment epithelial-19 (ARPE-19) cells and human Müller MIO-M1 cells (Salimiaghdam et al., 2020; Salimiaghdam et al., 2022). Anti-VEGF drugs (i.e., afibercept, ranibizumab, and bevacizumab) also up-regulate the mRNA expression of BCL-RAMBO, together with caspases 3 and 9, in ARPE-19 cells and MIO-M1 cells (Cáceres-del-Carpio et al., 2020; Hamid et al., 2021). These findings suggest that antibiotics or anti-VEGF drugs have a negative impact on retinal diseases by modulating BCL-RAMBO expression.

### Regulation of BCL-RAMBO by alternative splicing

Alternative splicing generates multiple transcript variants, which augments the functional diversity of translation products. The human BCL-RAMBO gene is located on chromosome 22q11.21 and is transcribed into many splicing variants. The longest human BCL-RAMBO transcript variant 1 (NM_015367.4) is composed of seven exons and encodes a polypeptide of 485 amino acids (isoform a), which contains all four BH motifs. Human BCL-RAMBO transcript variant 2 (NM_001270726.1) encodes the longest isoform b (a polypeptide of 509 amino acids), which contains the BH1, BH2, and BH3 motifs, but not the BH4 motif. Other transcript variants are shorter than isoform a, mostly due to the lack of exons or alternate exons.

In addition to these transcript variants, transcript variant BCL-RAMBO β, which contains a 98-bp Alu-like sequence, is detected in human adult tissues (e.g., the heart, lymph nodes, and cervix) (Yi et al., 2003). Alu elements are short and repetitive transposable elements that are found in the human genome (Deininger, 2011). The BCL-RAMBO β variant encodes a polypeptide of 104 amino acids, which possesses the BH4 motif, but lacks the BH1, BH2, and BH3 motifs due to the stop codon in the inserted sequence (Yi et al., 2003). The transfection of BCL-RAMBO β and BCL-RAMBO promote etoposide- and taxol-induced cell death (Yi et al., 2003).

Plant homeodomain-finger domain protein 5A (PHF5A) is a component of the splicing factor 3B (SF3B) complex and is involved in the regulation of pluripotency, cell differentiation, and tumorigenesis (Strikoudis et al., 2016; Wang et al., 2019). In human lung adenocarcinoma H1299 and A549 cells, the knockdown of PHF5A or a small molecule inhibitor of PHF5A (pladienolide) induces the alternative splicing of many cell cycle-regulated genes and apoptosis-associated genes, including BCL-RAMBO (Mao et al., 2019).

Circular RNAs (circRNAs) are a new class of RNA molecules that are generated by 3’ to 5’ end-joining as a novel type of alternative splicing, and regulate gene expression by multiple molecular mechanisms (Li et al., 2018). RNA-binding motif protein 20 (RBM20) is an RNA-binding protein with two zinc finger domains that regulates heart-specific alternative splicing (Watanabe et al., 2018). Thirty-eight circRNAs are differently expressed, 26 of which are down-regulated and 12 are up-regulated, in the hearts of RBM20-deficient mice and wild-type mice, and circBcl2l13 is one of the up-regulated circRNAs (Aufiero et al., 2018). The regulation of BCL-RAMBO expression by circRNAs is discussed in the next section.

### Regulation of BCL-RAMBO expression by microRNAs (miRNAs)

miRNAs are approximately 22-nt RNAs that play a critical role in the repression of their targets (Bartel, 2018). In several experimental models, miRNAs have been reported to module the expression of BCL-RAMBO (Table 3). The up-regulation of miR-21 down-regulates three putative novel target genes (*bcl2l13*, *rgs5*, and *chka*) in addition to two known target genes (*pdcd4* and *tgfbr2*) during limp/appendage blastema formation in Zebrafish, bichir, and axolotl, which provide highly regenerative model systems (King and Yin, 2016). These studies indicate that BCL-RAMBO expression is negatively regulated by multiple miRNAs in vertebrates, including mammals.

**TABLE 3:** Regulation of BCL-RAMBO expression by miRNAs.

miRNAs	lncRNAs/circRNAs	Experimental models	Tested species	Tested tissues/cell types	References
miR-21 miR-181c		Limb/appendage regeneration	Zebrafish	Limb/appendage blastoma	King and Yin (2016)
			Bichir		
			Axolotl		
miR-124 miR-137		Kainic acid-induced seizure	Mouse	Hippocampal neural stem/progenitor cells	Schouten et al. (2015)
				
miR-874-3p		OGD/R	Human	SH-SY5Y neuroblastoma cells	Jiang et al. (2019)
		I/R injury	Rat	Brain	
miR-765	FOXD3-AS1	OGD/R	Mouse	N2a neuroblastoma cells	Lu et al. (2020)
		I/R injury	Mouse	Brain	
miR-141-3p	SNHG15	Osteoarthritis	Human	IL-β-stimulated chondrocytes	Zhang et al. (2020)
			Human	HEK293T cells	
miR-526b-5p	Circ_0062166	OGD/R	Mouse	N2a neuroblastoma cells	Guo et al. (2021)
miR-1306-5p	H19	OGD/R	Human	SK-N-SH neuroblastoma cells	Huang et al. (2021)
			Human	IMR-32 neuroblastoma cells	
		I/R injury	Rat	Brain	
miR-484		OGD/R	Mouse	Cortical neurons	Liu et al. (2021)
miR-96-5p		Hypoxia	Rat	H9c2 cardiomyocytes	Ding et al. (2022)
miR-98-5p	SNHG14	OGD/R	Mouse	N2a neuroblastoma cells	Zhang et al. (2022)
		I/R injury	Mouse	Brain	

Abbreviations: I/R, ischemia/reperfusion; OGD/R, oxygen-glucose deprivation/reoxygenation.

The regulation of BCL-RAMBO expression by miRNAs plays a critical role in several experimental disease models. BCL-RAMBO promotes caspase 3 activity and CYCS release in mouse neural stem/progenitor cells (Schouten et al., 2015). The expression of miR-124 and miR-137 is up-regulated in a kainic acid-induced seizure model, which cooperatively reduces endogenous BCL-RAMBO expression and controls caspase 3 activity (Schouten et al., 2015). Cerebral ischemia/reperfusion (I/R) injury is a serious clinical condition that induces the production of reactive oxygen species and ultimately triggers cell death pathways (Wu et al., 2018). In addition to *in vivo* I/R injury animal models, the oxygen-glucose deprivation and reoxygenation (OGD/R) assay system has been used to examine the effects of miRNAs on BCL-RAMBO expression in *in vitro* cell cultures. The expression levels of miR-874-3p, miR-765, mi-526b-5p, miR-1306-5p, and miR-98-5p are reduced in OGD/R-induced neuroblastoma cells, which conversely up-regulates BCL-RAMBO expression, thereby promoting pathogenic changes, including apoptosis (Jiang et al., 2019; Lu et al., 2020; Guo et al., 2021; Huang et al., 2021; Zhang et al., 2022). The expression of miR-484 is down-regulated in mouse cortical neurons exposed to OGD/R, which promotes the expression of BCL-RAMBO (Liu et al., 2021). The expression of miR-141-3p down-regulates that of BCL-RAMBO in human IL-1β-stimulated chondrocytes (Zhang et al., 2020). In acute myocardial infarction associated with coronary artery disease, the expression of miR-96-5p is down-regulated, whereas that of BCL-RAMBO is up-regulated (Ding et al., 2022). Based on the pro-apoptotic effects of BCL-RAMBO, these findings show that the suppression of BCL-RAMBO expression is beneficial for therapeutic interventions.

Long non-coding RNAs (lncRNAs) and/or circRNAs have been reported to regulate BCL-RAMBO expression by targeting miRNAs. SNHG15 promotes BCL-RAMBO expression by sponging miR-141-3p in osteoarthritis cartilage tissue (Zhang et al., 2020). In I/R injury and ODG/R, lncRNAs (i.e., FOXD3-AS1, H19, and SNHG14) are up-regulated and promote the expression of BCL-RAMBO by sponging their target miRNAs (Lu et al., 2020; Huang et al., 2021; Zhang et al., 2022). Circ_0062166, generated from exon 3 of BCL-RAMBO, is up-regulated following OGD/R and regulates BCL-RAMBO expression by sequestering miR-526b-5p (Guo et al., 2021). These studies revealed that BCL-RAMBO expression is mainly regulated by the lncRNA/miRNA axis or circRNA/miRNA axis in I/R injury and osteoarthritis.

### A *Drosophila* model expressing human BCL-RAMBO

To the best of our knowledge, BCL-RAMBO homologues have not yet been reported in *Drosophila*. Human BCL-RAMBO induces apoptosis in *Drosophila* Schneider 2 cells by triggering the release of CYCS and caspase activation through its overexpression (Nakazawa et al., 2016). The ectopic expression of human BCL-RAMBO in *Drosophila* by the GAL4-UAS system manifests aberrant morphological changes, which are accompanied by the atrophied wing, split thorax, and rough eye phenotypes (Nakazawa et al., 2016). Among these changes, the rough eye phenotype induced by BCL-RAMBO is severe and characterized with a reduction in eye size, the loss of ommatidia and bristles, and the loss of pigmentation (Nakazawa et al., 2016). Caspase activation also occurs in eye imaginal discs in *Drosophila* expressing BCL-RAMBO (Nakazawa et al., 2016). The loss of pigmentation induced by the ectopic expression of BCL-RAMBO is partly suppressed by the co-expression of Diap1 and Diap2 as well as ANT mutations (Nakazawa et al., 2016). This is consistent with previous findings showing that BCL-RAMBO-induced caspase activity is suppressed by IAP proteins in 293T cells (Kataoka et al., 2001) and also that ANT is necessary for BCL-RAMBO-induced cell death (Kim et al., 2012). To identify BCL-RAMBO-interacting partner genes, human BCL-RAMBO-expressing fly lines have been crossed with fly lines with the deletion of or mutations in specific genes. Comprehensive screening using fly lines deficient for the second chromosome revealed that the BCL-RAMBO-induced rough eye phenotype is also partly suppressed by mutations in or the knockdown of porin and pgam5-2 (Nakazawa et al., 2016; Matsubara et al., 2019; Hashino et al., 2022). The genetic interaction of BCL-RAMBO with Porin or Pgam5 has been confirmed in human cultured cell lines. Therefore, the humanized *Drosophila* model expressing BCL-RAMBO may be a useful tool for identifying unknown partner genes.

### Conclusion and perspectives

BCL-RAMBO was initially identified as one of the BCL-2 family proteins inducing apoptosis (Kataoka et al., 2001). Approximately 15 years later, BCL-RAMBO has been shown to function as a mammalian Atg32 homologue that regulates mitophagy and mitochondrial fragmentation (Murakawa et al., 2015). In the 20 years since its first discovery, many important findings on the biological properties of BCL-RAMBO have been increasingly reported. BCL-RAMBO is characterized by one to four BH motifs and the LIR motif, and is anchored to MOM. BCL-RAMBO interacts with multiple proteins, including ANT, VDAC, MAP1LC3/GABARAP, ULK1, PGAM5, and CERS, thereby regulating cellular processes, including apoptosis, mitochondrial fragmentation, and mitophagy. BCL-RAMBO expression is regulated by multiple transcription factors, miRNAs, and lncRNAs. BCL-RAMBO is expressed in normal and cancer cells and has been implicated in pathogenic conditions, such as tumorigenesis and I/R injury. Nevertheless, the molecular mechanisms that link BCL-RAMBO to its binding partners, the regulation of its expression, and its physiological and pathological roles have not yet been elucidated in detail. Therefore, future studies are needed to address unanswered questions on BCL-RAMBO and other mitophagy receptors.
